# In-group biases and oculomotor responses: beyond simple approach motivation

**DOI:** 10.1007/s00221-018-5221-7

**Published:** 2018-03-07

**Authors:** Zahra Zargol Moradi, Sanjay Manohar, Mihaela Duta, Florence Enock, Glyn W. Humphreys

**Affiliations:** Cognitive Neuropsychology Centre, Department of Experimental Psychology, University of Oxford, New Radcliffe House, Walton Street, Oxford, OX2 6AE UK

**Keywords:** In-group biases, Out-group, Pro-saccade, Anti-saccade, Approach, Avoidance

## Abstract

An in-group bias describes an individual’s bias towards a group that they belong to. Previous studies suggest that in-group bias facilitates approach motor responses, but disrupts avoidance ones. Such motor biases are shown to be more robust when the out-group is threatening. We investigated whether, under controlled visual familiarity and complexity, in-group biases still promote pro-saccade and hinder anti-saccades oculomotor responses. Participants first learned to associate an in-group or out-group label with an arbitrary shape. They were then instructed to listen to the group-relevant auditory cue (name of own and a rival university) followed by one of the shapes. Half of the participants were instructed to look towards the visual target if it matched the preceding group-relevant auditory cue and to look away from it if it did not match. The other half of the participants received reversed instructions. This design allowed us to orthogonally manipulate the effect of in-group bias and cognitive control demand on oculomotor responses. Both pro- and anti-saccades were faster and more accurate following the in-group auditory cue. Independently, pro-saccades were performed better than anti-saccades, and match judgements were faster and more accurate than non-match judgements. Our findings indicate that under higher cognitive control demands individuals’ oculomotor responses improved following the motivationally salient cue (in-group). Our findings have important implications for learning and cognitive control in a social context. As we included rival groups, our results might to some extent reflect the effects of out-group threat. Future studies could extend our findings using non-threatening out-groups instead.

## Introduction

Many studies over the last few decades have shown that when we associate ourselves with a social group, we exhibit biases in favour of members of that group. Such biases have been termed the in-group bias or in-group favouritism effect (Amodio and Devine 2006; Hewstone et al. 2002; Molenberghs 2013; Moradi et al. 2015, 2016; Turner et al. 1979). Typically, such studies have shown that in-group stimuli are more salient, and can influence many different aspects of cognition including decision-making, attitudes, empathy and memory (Howard and Rothbart 1980; Islam and Hewstone 1993; Van Bavel et al. 2008; Xu et al. 2009). One proposed consequence of an in-group bias is to promote approach and inhibit avoidance motor responses. The notion that approach motor responses are facilitated by in-group stimuli has been investigated using different types of motor responses including an approach (pull) or an avoidance (push) hand motor action (Cunningham et al. 2012; Phills et al. 2011), showing facilitation of approach response towards the in-group targets.

However, some studies have used different types of motor responses and of note are the studies investigating the effect of in-group biases on oculomotor responses. In tasks involving oculomotor responses, participants are usually required to make an eye movement towards (pro-saccade) or away from (anti-saccade) an in-group or out-group target (Liuzza et al. 2011; Wu et al. 2012). For example, Liuzza and colleagues (2011) had participants make a pro-saccade congruent or incongruent with the direction of the gaze of a politician who either shared (in-group) or did not share (out-group) the same political viewpoints as those of the participant. For the individuals who shared the views of the politician, pro-saccades towards the target were disrupted when the gaze direction of the politician was incongruent with the instructed direction of the eye movement. Such an effect was not found for individuals who did not share the views of the politician. In-group biases can, therefore, modulate gaze-following approach oculomotor responses.

Previous studies have also examined the effects of in-group biases on avoidance oculomotor responses (Ciardo et al. 2013, 2014; Koval et al. 2005; Moradi et al. 2017; Payne 2005). For example, previous studies have shown that participants who made more errors on an anti-saccade task also showed a more significant implicit in-group bias as measured in a weapon identification task and a word evaluation task (Payne 2005, for more details, see; Greenwald et al. 1998). In another study, participants were instructed to either look towards or away from in- and out-group faces (Harvey et al. 2011). The results demonstrated that participants were slower and made more errors when looking away from in-group faces compared to out-group faces. These findings seem to suggest that there is a cost associated with efficient execution of making an anti-saccade away from the in-group target. Together, these results suggest that motivational salience of an in-group facilitates pro-saccades while disrupting or at least slows down anti-saccade oculomotor responses. However, two alternative interpretations could be considered.

First, in-group stimuli tend to be more familiar, leading to preferential processing. Familiarity could drive the salience effects of in-group observed for pro- (approach) vs. anti-saccade (avoidance) oculomotor responses. It has been shown that information that is processed faster (here, in-group relevant information) is more likely to trigger an orienting response (Mulckhuyse et al. 2009). Most previous studies used socially habituated stimuli such as faces or group symbols (see, for example, Moradi et al. 2017, as an example of non-face but socially habituated stimuli), which tend to show high in-group vs. out-group differences in the efficiency with which they are processed (Wu et al. 2012). Moreover, faces or other socially habituated stimuli do not easily allow controlling for visual properties of in- vs. out-group stimuli. The complexity and familiarity of the stimulus may fundamentally mask the actual effect of in-group biases on pro- and anti-saccade oculomotor responses. To overcome the confounding effects of visual familiarity and complexity in the present study, we used abstract shapes that had been previously associated with in-group and out-groups in a social associative learning task (Moradi et al. 2015).

A second interpretation relates to the different nature of pro- and anti-saccades. An anti-saccade response requires the suppression of a “reflexive pro-saccade” to permit an oculomotor response in the opposite direction of the visual target (Everling and Fischer 1998; Hallet and Adams 1980). The slower initiation of anti-saccades and the fact that anti-saccades are more error-prone than pro-saccades could be explained by the effort required to suppress the automatic pro-saccades or the extra cognitive control required to perform an anti-saccadic eye movement (Walker et al. 2000). Unlike pro-saccades, anti-saccade error rate and error-correction speed both correlate with working memory capacity (Kane et al. 2001). In line with this, the involvement of working memory in the execution of anti-saccadic eye movements has been shown by the data from a single case study of a patient with a frontal lobe lesion who made a high percentage of directional errors in the anti-saccade task and also showed deficits in working memory (Walker et al. 1998).

It is, therefore, possible that biases regarding pro- and anti-saccade responses, respectively, reflect facilitation of the easiest or most-natural response by in-group stimuli, or greater engagement of cognitive control processes by out-group stimuli. To address these issues, in this study we varied the cognitive control demands independently of pro- vs. anti-saccade oculomotor responses.

In the current study, we aimed to test whether in-group biases facilitate pro-saccade over anti-saccade oculomotor responses by controlling for familiarity and visual complexity of the targets. We used a novel paradigm that combines a social associative learning task with an eye-movement task. Participants first learned to associate in- or out-group labels (their own university or a rival university) with an arbitrary geometrical shape (for more details see, Moradi et al. 2015). They then viewed different pairs of labels and shapes and were required to indicate whether the pair they viewed was a match or a non-match based on what they had learned before. The use of abstract stimuli instead of visually complicated stimuli such as faces helped us to control for the visual familiarity as well as the visual complexity of the stimuli. To ensure that the participants learned the associations between the shapes and group labels only those participants with an overall accuracy above 70% in the social associative learning task proceeded to the eye-movement task. In the eye-movement task, participants were instructed to look towards or away from the visual targets (the same geometrical shapes were used in the social associative learning task) depending on whether or not they matched the preceding group-relevant auditory cue (the name of the in-group or out-group: own or rival university). In a between-subjects design, there were two versions of task instruction. In the “match-approach” version of the task, pro-saccades required lower cognitive control as the participants were instructed to look at the visual target if it matched the preceding auditory cue, and to look away from it if did not match. However, in the “match-avoid” version of the task, participants did the opposite, so that pro-saccades required higher cognitive control, as they were performed in response to a non-matched auditory cue.

We orthogonally manipulated the motivational salience of the auditory cue (in-group vs. out-group) and cognitive control demands required for each movement type (pro- vs. anti-saccade) while controlling for the familiarity and complexity of the visual target. We hypothesized that if the in-group works as a salient cue that facilitates a pro-saccade over an anti-saccade oculomotor response, then pro-saccade performance should be improved after the in-group auditory cue, whereas anti-saccade performance should be disrupted. However, if the in-group bias drives general motivation or improves cognitive control, then in-group auditory cues should also enhance performance in anti-saccade trials. This would manifest in reduced error rates and faster initiation of anti-saccades that follow the in-group auditory cue compared to the out-group cue.

## Method

### Participants

A total of 57 participants were recruited for this study. Three participants did not complete the session due to poor calibration, and another participant dropped out after the first block due to severe fatigue. The remaining 53 participants (13 male) were aged between 18 and 34, mean (SD) = 25.7 (4.0), right-handed, had normal or corrected-to-normal vision and reported no previous neurological or psychiatric conditions. Participants were recruited via an online advertisement. Before the experiment, all participants signed a written consent form approved by the University of Oxford research ethics committee.

### Stimuli

The target stimuli were square and diamond shapes, 50% grey with a solid black outline and 2° visual angle in size. The stimuli were presented in the middle of the screen at 9° of visual angle off-centre on the horizontal axis on a 50% grey background. The auditory stimuli were audio recordings of a male native English speaker pronouncing the single-word names of two rival (in-group and out-group) universities (Oxford and Cambridge).

### Procedure

**Fig. 1:**
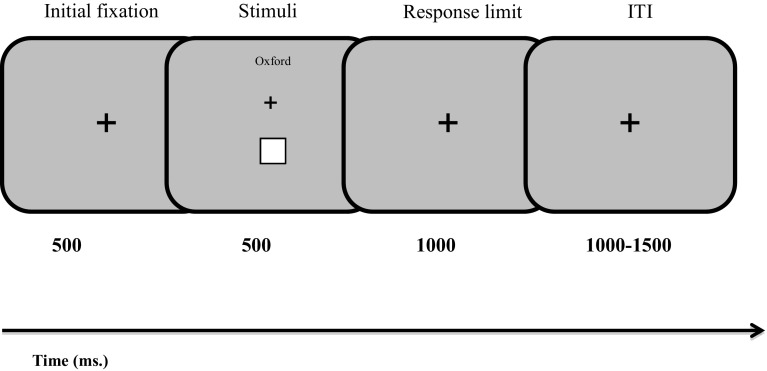
Schematic representation of the social associative learning task. The original background was 50% grey

The experiment comprised two tasks: a social associative learning task and an eye-movement task. Participants first learned to associate in-group and out-group visual labels with an arbitrary shape. For example, they learned to associate the in-group label with the square and the out-group label with the diamond. Then, participants viewed a shape–label pair and were instructed to make a response using the keyboard indicating whether the pair was a match or a non-match based on the association that they just learned. Participants were given feedback after each trial stating that their response was “correct”, “incorrect” or “slow”. The range of RTs that were included for the ‘slow’ feedback was between 1100 and 1500 ms. At the end of the associative learning task, participants were given feedback on their overall accuracy. To ensure that the participants learned the associations between the shapes and in- and out-group labels, only those with an overall accuracy higher than 70% in the associative learning task were included in the eye-movement task. A schematic representation of the social associative learning task can be found in Fig. 1.

**Fig. 2:**
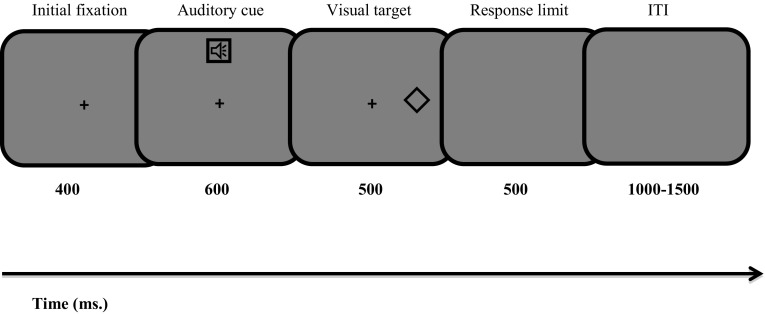
Illustration of the eye-tracking task. The original background was 50% grey

In the eye-movement task, participants were presented with a spoken audio label of in- and out-group universities (“Oxford” or “Cambridge”) followed by a visual target (the same geometrical shapes that were used in the social associative learning task). They were instructed to make a pro-saccade or an anti-saccade oculomotor response depending on whether or not the visual target matched the preceding group auditory cue according to the rules they had learned previously in the social associative learning task. Participants were randomly assigned into two groups, with each group performing either the high- or the low-demand version of the eye-movement task. In the low-demand version of the task, participants were instructed to make a pro-saccade to the visual target if it matched the preceding auditory cue and to make an anti-saccade away from it if it did not match the preceding auditory cue. For example, if participants learned to associate the diamond with “Oxford” (in-group auditory cue), then every time they heard the word “Oxford” they should have made a pro-saccade towards the diamond if it appeared next. However, if they heard “Oxford” and then the square appeared, they should have made an anti-saccade away from the shape. A schematic representation of the eye-movement task can be found in Fig. 2.

In the high-demand version of the task, participants were instructed to do the opposite and make a pro-saccade toward the visual target if it did not match the preceding auditory cue and make an anti-saccade away from the visual target if it did match the preceding auditory cue. It is reasonable to suppose that the latter version of the task is more demanding in terms of required cognitive control. For example, participants needed extra cognitive control to look away from the visual target that matched the preceding auditory cue instead of looking at it. Also, it could be argued that a pro-saccade is a more readily available (prepared) action if the visual target matches the proceeding auditory cue (congruency between match judgement and the required eye movement). If this is true, then participants would make more directional errors in the highly demanding version of the task on pro-saccade trials compare to the low demanding version of the task. Such a contrast between the two versions of the task allowed us to better understand the motivational effects of in-group on pro-saccade vs. anti-saccade oculomotor responses under different levels of task difficulty (cognitive control demand).

### Apparatus

The experiment was implemented and run in Matlab (32-bit version R2012a; The MathWorks, Natick, MA, USA) with Psychophysics Toolbox (Brainard 1997). Participants sat approximately 58 cm away from a 22-inch screen (51 × 32 degree of visual angle, 1920 × 1080 pixels, 100 ppi, 60 Hz refresh rate) in a normally lit room with their chin on a chinrest. Eye movements and pupil diameter were recorded from both eyes using Tobii eye tracking equipment (TX300, Tobii, Stockholm) running at 300 Hz. Prior to the test session, a nine-point calibration procedure was conducted, with a spinning ball target of 2° of visual angle in size. During calibration, participants were instructed to follow the ball with their eyes (without moving their head) and to fixate on the ball every time it stopped at each of the nine locations. Calibration was repeated up to three times if the participant failed to fixate on the target on fewer than seven points out of nine. The experiment did not continue if the calibration failed after three attempts.

Each trial started with a black fixation cross (1° visual angle) at the centre of the screen for a minimum length of 500 ms. If the participant fixated within a 2° visual angle area around the fixation cross for at least 100 ms, then the trial proceeded. However, if the participant failed to fixate in the first attempt, the fixation presentation was repeated up to two more times. If the participant failed to fixate after three attempts, the trial was skipped and flagged as invalid.

The trial continued with an auditory cue for 600 ms, during which participants were required to keep fixating on the central cross. The visual target then appeared randomly to the left or right of the fixation cross for 500 ms and then a blank screen was presented for 500 ms. A random inter-trial interval (ITI) of 1000 to 1500 ms was inserted after each trial (Barton et al. 2006).

Each participant was presented with 3 blocks of 80 mixed pro- and anti-saccade trials, for a total of 240 trials. Pro-saccade and anti-saccade trials were presented in a random order prior to the test block; there was a short practice block (8 trials) for the participants to become familiar with the task. Each participant was tested in one experimental session that lasted for approximately 45 min with the option of taking up to a 5-min break after each block.

## Results

Descriptive statistics for both versions of the task can be found in Table 1.

**Table 1:** Mean (SD) characteristics of correct pro- and anti-saccades for the match-approach (low cognitive control demand) and match-avoid (high cognitive control demand) versions of the eye-movement task

Task version	Directional error, %	Latency, ms	Amplitude, degree visual angle
Low demand (match-approach)			
Pro-saccade			
In-group	0.06 (0.01)	293 (66)	8.74 (0.85)
Out-group	0.08 (0.01)	302 (58)	8.73 (0.91)
Anti-saccade			
In-group	0.47 (0.03)	473 (189)	7.65 (2.63)
Out-group	0.51 (0.03)	476 (211)	7.55 (2.77)
High demand (match-avoid)			
Pro-saccade			
In-group	0.17 (0.01)	409 (108)	8.45 (1.16)
Out-group	0.19 (0.02)	423 (111)	8.14 (1.15)
Anti-saccade			
In-group	0.36 (0.03)	463 (99)	8.40 (2.50)
Out-group	0.41 (0.03)	481 (102)	8.20 (2.16)

Gaze data were processed using custom Matlab scripts (for the pre-processing parameters see, DeSimone et al. 2015; Everling and Fischer 1998). We removed the multi-step eye-movement responses from the analyses. Corrective saccades were not included in the analyses and statistics were performed in SPSS. For all analyses, a mixed-effects 2 × 2 × 2 ANOVA was performed, with the within-subject independent variables of the required movement type (pro-saccade vs. anti-saccade) and group relevance of the auditory cue (in-group vs. out-group) and the between-subject variable of the task type (High vs. Low demand)^1^. Post hoc t tests were then performed to describe key findings in more detail. The details regarding statistical analyses can be found in Table 2.

**Table 2:** Results of the mixed three-way ANOVA showing the effects of independent variables on different metrics of oculomotor responses

Oculomotor metrics	Main effects *F*(1,51)			Interactions *F*(1,51)			
	M	G	T	MT	MG	GT	MGT
Directional error	111.11***	12.23***	0.004	12.58***	1.77	0.158	0.005
Latency	40.91***	6.33*	3.78	11.06**	0.006	1.20	0.379
Amplitude	4.08*	4.68*	0.417	4.17*	0.445	0.038	1.29

Within-subject variables included: 1-movement (M): pro-saccade/anti-saccade, 2-group relevance of auditory cue (G): in-group/out-group. Between subject variable included: 1. Task type (T): match-approach/match-avoid

*p < 0.05, **p < 0.01, ***p < 0.001

### Directional errors

We first quantified how often participants made errors by looking to the wrong side of the screen (directional errors). Our results revealed a significant main effect of group relevance of the auditory cue on the percentage of directional error. On average, participants made fewer directional errors following the in-group auditory cue compare to that of the out-group. There was also a main effect of movement type, with more errors in anti-saccade compared to pro-saccade oculomotor responses. There was a significant interaction between movement type and task type, with larger pro- vs. anti-saccade differences evident in the low-demand version of the task (match-approach) [*t*(26) = 10.45, *p* < 0.001, *d* = 4.09; mean difference ± SD = 0.417 ± 0.039] compared to the high demand (match-avoid) version of the task [*t*(25) = 4.72, *p* < 0.001, *d* = 1.70; mean difference ± SD = 0.207 ± 0.043]. Compared to those who took part in the low-demand version of the task, participants who took part in the high-demand version of the task made more directional errors in pro-saccade responses [*t*(25) = 4.55, *p* < 0.001, *d* = 1.81; mean difference ± SD = 0.104 ± 0.022]. An opposite pattern was observed for the anti-saccade responses showing that compared to those who did the low-demand version of the task, participants who performed the high-demand version of the task made significantly fewer directional errors in the anti-saccade oculomotor responses [*t*(25) = 2.09, *p* < 0.047, *d* = 0.83; mean difference ± SD = 0.109 ± 0.05]. It was noted that compared to the low-demand version of the task, in the high demand task the error rate was increased (by about 10%) for the pro-saccade responses. Interestingly, the increase in error rates for pro-saccades was comparable to the decrease in directional errors for anti-saccades. The interaction between the movement type and group relevance of the auditory cue was not significant; nor was the interaction between group relevance of the auditory cue and task type; and nor was the three-way interaction of movement type, group relevance, and task. See Table 2 for details on statistical analyses.

### Latency

Next, we examined the time that participants took to initiate a correctly directed eye movement to investigate whether there was an effect of movement type, group relevance of the auditory cue as a within-subject, or task type as a between-subjects, variable. Our results demonstrated a main effect of group relevance of the auditory cue on the latency of correct saccades. On average, participants were faster to start a correct pro-saccade, or an anti-saccade response following the in-group auditory cue compared to the out-group auditory cue, supporting the hypothesis of the motivating effect of in-group. There was a significant main effect of movement type, with participants being faster to initiate a pro-saccade compared to an anti-saccade oculomotor response.

The main effect of task type on the latency of correct saccades was not significant. However, the interaction between movement type and task was significant. The difference between the latency for pro- vs. anti-saccade oculomotor response was larger in the low demand (match-approach) version of the task [*t*(26) = 5.49, *p* < 0.001, *d* = 2.15; mean difference ± SD = 176.77 ± 32.19] compared to the high demand (match-avoid) version of the task [*t*(26) = 5.49, *p* < 0.001, *d* = 2.15; mean difference ± SD = 55.80 ± 15.94]. Participants were significantly slower to initiate a pro-saccade response towards the visual target if it did not match the preceding auditory cue (high demand task) compared to when the visual target matched (low demand task) the preceding auditory cue [*t*(25) = 4.88, *p* < 0.001, *d* = 1.95]. No significant difference was found on the latency of the anti-saccade response between the two versions of the task [*t*(25) = 0.159, *p* < 0.875]. Neither the interaction between group relevance and movement type nor the interaction between group relevance and task was significant; and the three-way interaction between movement type, group relevance, and cognitive control demand was also not significant.

### Amplitude

On trials for which the correct direction of the eye movement was selected, motivational effects on the action could be observed in more detail by examining the amplitude of the saccade. Since saccades normally land short of a target (i.e., they tend to be hypometric), larger amplitudes indicate a more strongly motivated movement that lands closer to the target (Manohar et al. 2015).

Our results revealed that there was a significant main effect of group relevance on the amplitude of the saccades. On average, pro-saccades were larger following the in-group auditory cue compared with that of the out-group. There was also a main effect of movement type. On average pro-saccades were larger than the anti-saccades. This difference was affected by the task type, but not by group relevance. In the low demand (match-approach) version of the task, the amplitude of pro-saccades was larger than the amplitude of anti-saccades [*t*(26) = 2.62, *p* < 0.014, *d* = 1.02]. However, in the high demand (match-avoid) task, the amplitude of pro-saccades did not differ compared with the amplitude of anti-saccades [*t*(25) = 0.018, *p* < 0.986].

The main effect of task type on the amplitude of correct saccades was not significant. The interaction between group relevance and movement type was also not significant, and nor was the interaction between group relevance and task type. There was also no significant three-way interaction. Detailed statistical analyses can be found in Table 2.

## Discussion

In the current study, we investigated the effects of in-group biases on oculomotor responses. Previous findings suggest that in-group biases facilitate pro-saccades while hindering or slowing down anti-saccade oculomotor responses (Ciardo et al. 2013; Liuzza et al. 2011; Moradi et al. 2017). We argued that such effects might be driven by the higher familiarity of the in-group target. More importantly, confounding factors such as the complexity of the visual targets and the different level of execution difficulty regarding oculomotor responses (anti- vs. pro-saccades) could have affected the previous findings. Pro-saccades are automatic and more natural responses than anti-saccades, which are harder to execute and demand more cognitive control. To reduce these confounds, we examined the relationship between in-group biases and pro- vs. anti-saccade oculomotor responses, controlling for the familiarity and visual complexity of the targets. Furthermore, we orthogonally manipulated the need for cognitive control for each type of motor response. We asked whether the in-group association specifically facilitates pro-saccades and in turn disrupts anti-saccades. Moreover, we introduced two levels of task demand to test how the need for additional cognitive control might affect the natural, automatic pro-saccade as opposed to the more cognitively demanding and inhibitory anti-saccade responses.

Our study provides evidence for the effects of an in-group bias on both pro- and anti-saccade oculomotor responses. Our results indicate that when controlled for familiarity and visual complexity, the in-group bias does not merely enhance performance in pro-saccades and nor does it hinder anti-saccades. Our findings rather imply that in-group bias improves performance in both pro- and anti-saccades. This was revealed through the analyses of three important metrics of oculomotor responses including percentage of directional error, latency, and amplitude of eye movements.

Our results demonstrated that participants made fewer directional errors following an in-group auditory cue. This was the case for both pro- and anti-saccade oculomotor responses. The directional error is an important indicator of cognitive control especially in anti-saccade movement (Hallett and Adams 1980). Previous studies have shown that social and motivational factors including group identification may affect the directional errors in anti-saccade oculomotor responses (Manohar et al. 2015; Moradi et al. 2017). This could be specifically the case when the stimuli are related to real-world intergroup conflicts. For example, in our previous study (Moradi et al. 2017) we used the badges of an in-group football team, a traditional rival team and a familiar but neutral (no historical rivalry) team. We found that participants made more directional errors on anti-saccade trials for the badge of their favourite team and in turn fewer errors when looking away from the badge of the rival team. The findings of our current study are not in line with previous studies (Luizza et al. 2011; Moradi et al. 2017), including our own previous research. Such a discrepancy between our current findings and the previous findings could be because of the different stimuli used in previous studies compared with the stimuli that we used. Moreover, since cognitive control demand was not manipulated in the previous studies, a higher rate of directional error was expected for the anti-saccade trials compared with the pro-saccade trials. As we manipulated cognitive control demand in our present study, this made both pro- and anti-saccade motor responses prone to the directional error depending on the cognitive control demand required for each type of movement. Furthermore, the different contexts in which in-group effects on oculomotor responses were tested could result in the inconsistency in our findings compared with previous findings. Our results also revealed that for both pro- and anti-saccades, participants were faster to initiate the eye movement in the correct direction following the in-group auditory cue compared with the out-group auditory cue. The third significant finding of our study was that the amplitude of both pro- and anti-saccades was larger following the in-group auditory cue, resulting in eye fixation landing closer to the location of the target.

We further found that for both pro- and anti-saccades, participants were faster and more accurate when a match judgement, compared to a non-match judgement, had to be made. Notably, there were no interactions of group relevance with the type of judgement, indicating that the effect of in-group bias was independent of cognitive control demand. We were still able to show that in-group bias (favouritism) improved performance as revealed in directional error, the latency of initiation of the movement as well as the amplitude (accuracy) of the movement, even though the effects were small and did not interact with other variables.

With regards to the effect of movement type, we found that pro-saccade responses were faster and more accurate, especially in easier response mapping compared to the more laborious mapping (match vs. non-match) and this was independent of the in-group bias effects on eye movements. Our results further showed that the performance was also affected by the task type. Both anti-saccades and non-match judgements were more difficult to make compared to pro-saccades and match judgements, respectively.

Overall, our results showed that an in-group bias impacted on essential metrics of the saccadic eye movements including directional errors, latency, and amplitude. In real life situations, such effects could have a profound impact on intergroup interactions. For example, in conflicting situations, slower latency and smaller amplitude of eye movements may result in paying less attention to the members of an out-group who might be in need of help.

Previous studies have shown that activation of approach and avoidance motor responses could be affected by the motivational effects of in-group relevance. The findings of those studies suggest that in-group relevance might trigger approach (pro-saccades) and in turn hinder avoidance (anti-saccades) visual motor responses (Ciardo et al. 2013; Payne 2005). Here, controlling for familiarity and visual complexity, our study provides further evidence indicating that previous findings might be affected by the higher level of familiarity of in-group targets, and that anti-saccade visual motor responses require greater cognitive control. Our findings are in line with those of Koval and colleagues (2005) who used schematic faces and showed that task instruction could affect cognitive control and improve performance independent of social factors such as gaze direction.

Our findings are also consistent with previous research showing that motivational factors such as group identification could modulate performance in eye-movement tasks (Liuzza et al. 2011; Payne 2005; Wu et al. 2012). Our study is the first to use different levels of task cognitive demand orthogonally to group identification effects. Such a manipulation helped to better understand the motivational effects of in-group on oculomotor responses. Adding an extra level of cognitive control demand allowed us to examine the extent of in-group motivational effects on oculomotor responses. Our findings indicate that when additional computations are needed to control the oculomotor response, individuals’ performance improved following the motivationally salient cue (in-group). Such an effect might indicate that an in-group bias (even in an abstract form) works as a positive motivational cue similar to reward. Indeed, in agreement with this interpretation, previous studies have found that reward (also in an abstract form) can enhance performance in eye-movement tasks (Manohar et al. 2015).

However, in our study the rivalry between the two groups (Oxford and Cambridge) might have resulted in enhanced performance for the in-group in the eye-movement task. Importantly, we showed that the motivational effects of group were not limited to pro-saccades. These findings suggest that under well-controlled conditions and across different levels of difficulty, the motivational salience of in-group can result in enhanced speed and accuracy for both pro- and anti-saccades. Our findings are both surprising and novel with regard to the results of similar previous research, which indicate the disruption of anti-saccades away from in-group faces (Ciardo et al. 2013). Our results suggest that higher familiarity of in-group faces might at least partially account for previously observed disruption in anti-saccade performance, resulting in a higher rate of directional errors towards the in-group faces. Our findings are important as they imply that in a real social context, familiarity might drive many of the biases we have towards the members of our own age, gender, and race. Therefore, it might be possible to reduce the effects of familiarity by increasing intergroup contact. Further studies could provide evidence on the effects of intergroup contact on pro- and anti-saccades.

The oculomotor response is a type of adaptive behaviour that could be affected by different internal and contextual factors, including in-group biases. Individuals need to optimally control their eye movements in order to successfully monitor the changing environment for motivationally salient targets. Social factors such as an in-group bias may enhance the saliency of the target and therefore attract visual attention (see, for example, Van Bavel et al. 2008). However, in real life situations this does not necessarily mean that the in-group target is always higher in saliency. Studies have shown that under certain conditions, such as threat, an out-group might become more salient than an in-group (see, Capellini et al. 2016; Hackel et al. 2014) and therefore enhanced visual monitoring of the out-group target occurs. It is worth noting that in the context of our study, improved performance following the motivational (in-group) cue could be interpreted as higher saliency for in-group. Nevertheless, the saliency of the in-group could have been affected by the rivalry between the two target groups in our study. Such salience might improve motivation or arousal, without necessarily invoking attentional capture or triggering approach behaviour.

## Limitations

Although we made every effort to control for different confounds affecting our findings, there remain some shortcomings. In our study, the intergroup context in the form of the rivalry between Oxford and Cambridge might have had some impact on the salience of the in-group vs. out-group stimuli. We used intergroup rivalry to establish group-relevant salience. It is worth noting that the intergroup rivalry might differentially affect the motivational salience of in-group vs. out-group. Future studies might start to investigate how in-group biases affect oculomotor responses in the absence of intergroup rivalry. One way to conduct such studies is to use a minimal group paradigm (Brewer 1979), in which intergroup rivalry could be set at the minimum level.

Another limitation of the current study was that our participant sample was not balanced with regards to gender ratio (there were more female than male participants). Considering that we used a male speaker for the group-relevant auditory cue, such a difference might have had a confounding effect on our results. It would be ideal for the future studies to use a balanced sample of both genders. We would also like to indicate that, in our study, the group information was highly relevant to the eye-movement task. It would be interesting for the future studies to test whether making the group information irrelevant to the eye-movement task can still have an impact on participants’ looking behaviour.

Last but not least, we would like to acknowledge that the effect sizes of the in-group biases on saccadic metrics were small, yet still significant. We argue that this might at least to some extent be related to the design of the social associative learning task that was conducted prior to the eye-movement task. In the social associative task, participants learned to associate in-group and out-group visual labels with an arbitrary geometrical shape. This association was a unimodal association (visual), while in the eye-movement task, participants were required to translate the group-relevant cue from visual modality to auditory modality (cue) and then judge whether the following visual target matched this auditory cue. Such cross-modal translation might have made the information processing in the eye-movement task more difficult and therefore resulted in a smaller effect of in-group biases on pro- and anti-saccades. This issue could be further investigated in future studies.

## Footnotes

1 In the low-demand (match-approach) version of the task, participants were instructed to make pro-saccades towards the visual target when it matched the preceding auditory cue and anti-saccades away from it when it did not match the preceding auditory cue. This was reversed in the high-demand (match-avoid) version of the task.
